# How I do it: implantation of Osia® 2 system under local anesthesia

**DOI:** 10.1007/s00405-024-08921-5

**Published:** 2024-09-06

**Authors:** Bálint Posta, László Rovó, Zsófia Bere

**Affiliations:** Department of Oto-Rhino-Laryngology and Head-Neck Surgery, University of Szeged, Szeged, Hungary

**Keywords:** Bone conduction hearing, Auditory prosthesis, Osia, Local anesthesia

## Abstract

**Introduction:**

Reviewing the literature, Osia 2 system implantation is predominantly performed under general anesthesia (GA). Although in the pediatric population GA is inevitable, in adult cases, especially with high anesthesiological risks, local anesthesia (LA) is an obvious solution.

**Method:**

The aim of this article is to provide a detailed demonstration of Osia 2 implantation under LA. In our case series of five adult implant recipients, the surgical procedure was carried out without encountering any difficulties during or after the operation.

**Conclusion:**

Based on our experiences, implantation of the Osia® 2 System under local anesthesia is an easy and safe method for patients.

## Introduction

**Fig. 1:**
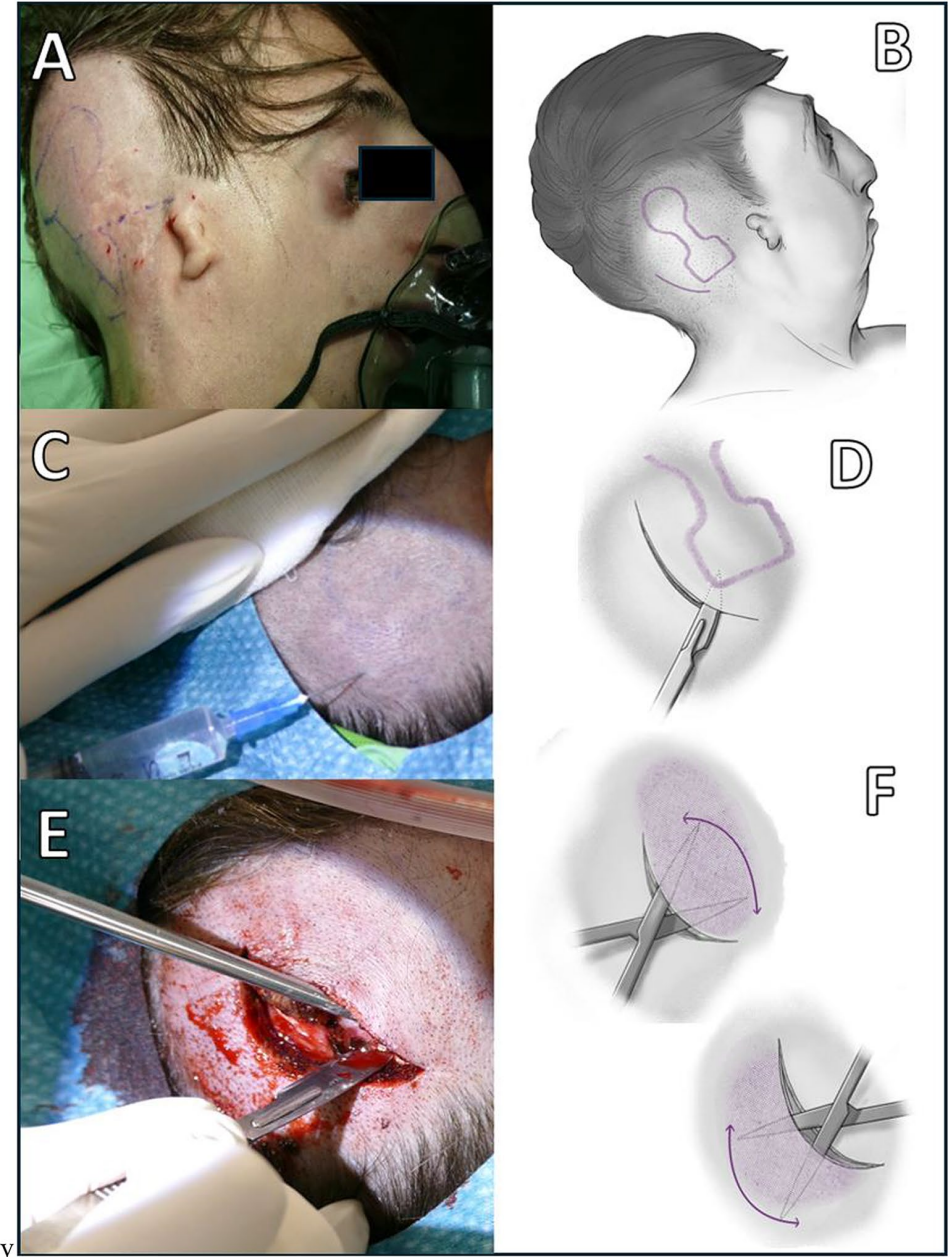
Surgical steps with schematic drawing of the Osia 2 system implantation under local anesthesia in adult patient with Treacher-Collins syndrome: **A-B** Determine the precise location for creating the incision and placing of OSI200 and BI300 on patient. **C** Local infiltration of the region. **D** The incision line 1.5 cm away from the lower posterior edge of the transducer. **E** 5–6 cm arc-shaped incision was performed and **F** Elevation of the soft tissue with scissors and raspatory instrument, the flap creation in the region of the implant as well in the opposite (postero-inferior) direction

Osia has gained increasing popularity due to its exceptional performance and relatively straightforward surgery. Currently, all the 12 Osia recipients in Hungary (7 pediatric and 6 adult) were implanted in our clinic, and initial experiences with the surgery were reported by the group [1]. From the beginning, we encountered the need for implantation under local anesthesia (LA); intubation or the use of a laryngeal mask in one of our first adult patient with Treacher-Collins syndrome could prove challenging due to the patient’s severe craniofacial malformation and very limited mouth opening, and the patient declined the option of a tracheotomy (Fig. 1A). Therefore, standardized steps of Osia implantation under LA were developed.

## Materials and methods

Participants provided their informed consent by signing a form that was approved by the local institutional review board (Human Investigation Review Board, University of Szeged, Albert Szent-Györgyi Clinical Centre; number: 163/2020-SZTE).

Overall, 5 out of 6 adult patients were unilaterally implanted with Osia 2 system under LA (2 female, 3 male, aged 42 ± 7 years).

All patients received premedication consisting of an intramuscular injection of 1 mg atropine, 5 mg midazolam, and 20 mg nalbuphine hydrochloride, administered in combination 20–30 min prior to surgery. For postoperative pain management, a paracetamol 10 mg/ml 50 ml infusion (PARACETAMOL) or diclophenac-orphenadrine 75 mg/30 mg/250 ml (NEODOLPASSE) was administered.

### Surgical technique step by step

To ensure continuous monitoring of the patient, a pulse oximeter and a three-wire lead electrocardiogram were attached to the patient. A supplementary oxygen source was administered through either a nasal cannula or a facial mask (Fig. 1A).

**Fig. 2:**
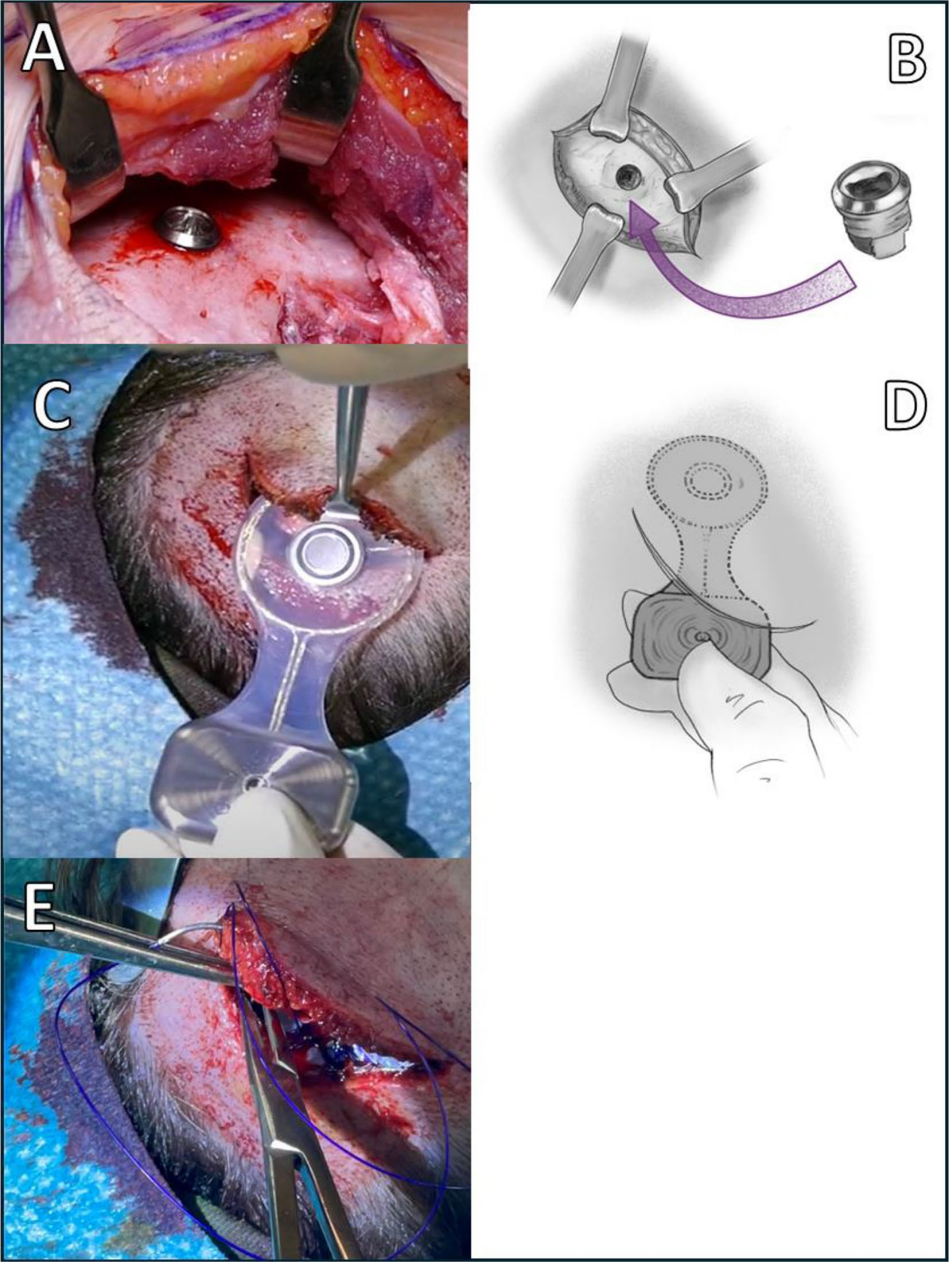
Next steps of Osia 2 system implantation surgery under local anesthesia: **A-B** After drilling the bone bed, the BI300 is implanted. **C-D** The OSI200 implant placing in the elevated pocket **E** Closing the wound

The Osia template was used to accurately determine the precise location for creating the incision and placing of OSI200 and BI300. The optimal placement of the transducer is approximately 1–1.5 cm above the midline of the ear canal, similar to the method described by Deep et al., where the flat surface of the bone allows for improved alignment without the need for additional bone removal and provides less protrusion and consequent tension on the sutures [2]. Additionally, the OSI200 implant was tilted anteriorly by approximately 20–25° around its axis in order to get the optimal position for the sound processor. The local infiltration procedure involved using a solution containing 10 ml of lidocaine (20 mg/ml), 0.2 ml of adrenaline (1 mg/ml), and 10 ml of saline. The solution infiltrated the predetermined region, and directly on the top of the bone, acting as a hydrodissector to facilitate the lifting of the periosteum (Fig. 1C). After 5 min, an approximately 5–6 cm arc-shaped incision was performed 1.5 cm from the lower posterior edge of the transducer (Fig. 1D-E). A direct and precise separation of the soft tissue flap was carried out using a scissors and raspatory instrument to establish a space for the OSI200 (Fig. 1F). As a mandatory step, soft tissue was also raised on the other side of the incision line to aid the manipulation with the Langenbeck retractor and later minimize the tension on the suture line. This incision type, together with the extended elevation of the temporal flap, allowed enough space to work comfortably with the Osscora™ Surgical Set to drill the bone bed (Fig. 2A-B). The BI300 fixed, and the OSI200 implant mounted on it (Fig. 2C-D). In one case, where soft tissue reduction was necessary at the level of the coil, a tunnel was created between the temporal and the temporoparietal fascia, and the coil part was inserted between these layers without the necessity of removing subcutaneous fat, while the implant body remained under the periosteum pocket. The musculo-periosteal layer was sutured with hand-tied 2–0 vicryl sutures due to the requirement for sutures capable of withstanding higher stress in this tightly layered area (Fig. 2E). Subsequently, the profound dermal layer and skin were closed in the customary manner. A mastoid dressing was placed on implant area.

## Results

The average surgical time was 27 ± 5 min (maximum 35 min). No massive intraoperative bleeding, dura injury, mastoid air cell exposure, or sigmoid sinus exposure was detected. No patients reported experiencing significant pain or discomfort during or after the surgery. There were no further issues related to wound healing or noticeable protrusions at the level of the transducer following the surgeries.

## Discussion

Implanting the Osia 2 system under LA can serve as a viable option for patients who face a significant risk of complications from general anesthesia, such as those with a challenging airway or a high ASA score. When comparing experiences with cochlear implantation (CI), CI surgeons tend to prefer using LA in cases where patients have comorbidities that make general anesthesia risky. Factors such as the availability of anaesthesiologist and the financial consequences might also play a role in this decision [3]. It is crucial to highlight that Osia 2 system has dimensions that make the size of the incision and the manipulation of soft tissue more similar to CI; however, it requires only minimal bonework—if it is necessary—compared to other bone-conductive solutions, especially in the modified position presented in our case. Although, proper planning, precise incision and extended flap undermining are necessary due to the thickness and size of the transducer. With tunnel technique, even soft tissue thickness problems in the coil region can be easily handle without the necessity of enlarge the approach. The average surgical time in our patient series also demonstrates that the procedure could be efficiently tolerated under LA, since the effect of the lidocaine infiltration last for 60–90 min, and the preoperative analgosedatives provide anxiolytic-hypnotic effect for 3–4 h. These several aspects contribute to the procedure being viable with the use of local anesthetic.

## Conclusion

Implantation of the Osia® 2 System under local anesthesia proves to be an easy and safe method for patients, offering a viable alternative to general anesthesia.

## Limitation

Implantation under LA is not recommended in children.
